# PEAL Score to Predict the Mortality Risk of Cardiogenic Shock in the Emergency Department: An Observational Study

**DOI:** 10.3390/jpm13111614

**Published:** 2023-11-16

**Authors:** Jen-Wen Ma, Sung-Yuan Hu, Ming-Shun Hsieh, Yi-Chen Lee, Shih-Che Huang, Kuan-Ju Chen, Yan-Zin Chang, Yi-Chun Tsai

**Affiliations:** 1 Department of Emergency Medicine, Taichung Veterans General Hospital, Taichung 407219, Taiwan; horseword70@gmail.com (J.-W.M.); ckz01119@gmail.com (K.-J.C.); rosa87324@gmail.com (Y.-C.T.); 2 Department of Post-Baccalaureate Medicine, College of Medicine, National Chung Hsing University, Taichung 402, Taiwan; 3 Institute of Medicine, School of Medicine, Chung Shan Medical University, Taichung 40201, Taiwan; 4 School of Medicine, Chung Shan Medical University, Taichung 40201, Taiwan; cucu0214@gmail.com; 5 School of Medicine, National Yang Ming Chiao Tung University, Taipei 11217, Taiwan; edmingshun@gmail.com; 6 Department of Emergency Medicine, Taipei Veterans General Hospital, Taoyuan Branch, Taoyuan 330, Taiwan; 7 Department of Emergency Medicine, Taipei Veterans General Hospital, Taipei 11217, Taiwan; leeyichen9@yahoo.com.tw; 8 Department of Emergency Medicine, Chung Shan Medical University Hospital, Taichung 40201, Taiwan; 9 Lung Cancer Research Center, Chung Shan Medical University Hospital, Taichung 40201, Taiwan; 10 Center for Cardiovascular Medicine, Taichung Veterans General Hospital, Taichung 407219, Taiwan; 11 Department of Clinical Laboratory, Drug Testing Center, Chung Shan Medical University Hospital, Taichung 40201, Taiwan

**Keywords:** cardiogenic shock, acute myocardial infarction, mortality risk, score, platelet counts

## Abstract

Background: The in-hospital mortality of cardiogenic shock (CS) remains high (28% to 45%). As a result, several studies developed prediction models to assess the mortality risk and provide guidance on treatment, including CardShock and IABP-SHOCK II scores, which performed modestly in external validation studies, reflecting the heterogeneity of the CS populations. Few articles established predictive scores of CS based on Asian people with a higher burden of comorbidities than Caucasians. We aimed to describe the clinical characteristics of a contemporary Asian population with CS, identify risk factors, and develop a predictive scoring model. Methods: A retrospective observational study was conducted between 2014 and 2019 to collect the patients who presented with all-cause CS in the emergency department of a single medical center in Taiwan. We divided patients into subgroups of CS related to acute myocardial infarction (AMI-CS) or heart failure (HF-CS). The outcome was all-cause 30-day mortality. We built the prediction model based on the hazard ratio of significant variables, and the cutoff point of each predictor was determined using the Youden index. We also assessed the discrimination ability of the risk score using the area under a receiver operating characteristic curve. Results: We enrolled 225 patients with CS. One hundred and seven patients (47.6%) were due to AMI-CS, and ninety-eight patients among them received reperfusion therapy. Forty-nine patients (21.8%) eventually died within 30 days. Fifty-three patients (23.55%) presented with platelet counts < 155 × 10^3^/μL, which were negatively associated with a 30-day mortality of CS in the restrictive cubic spline plot, even within the normal range of platelet counts. We identified four predictors: platelet counts < 200 × 10^3^/μL (HR 2.574, 95% CI 1.379–4.805, *p* = 0.003), left ventricular ejection fraction (LVEF) < 40% (HR 2.613, 95% CI 1.020–6.692, *p* = 0.045), age > 71 years (HR 2.452, 95% CI 1.327–4.531, *p* = 0.004), and lactate > 2.7 mmol/L (HR 1.967, 95% CI 1.069–3.620, *p* = 0.030). The risk score ended with a maximum of 5 points and showed an AUC (95% CI) of 0.774 (0.705–0.843) for all patients, 0.781 (0.678–0.883), and 0.759 (0.662–0.855) for AMI-CS and HF-CS sub-groups, respectively, all *p* < 0.001. Conclusions: Based on four parameters, platelet counts, LVEF, age, and lactate (PEAL), this model showed a good predictive performance for all-cause mortality at 30 days in the all patients, AMI-CS, and HF-CS subgroups. The restrictive cubic spline plot showed a significantly negative correlation between initial platelet counts and 30-day mortality risk in the AMI-CS and HF-CS subgroups.

## 1. Introduction

Cardiogenic shock (CS), a heterogeneous clinical syndrome with two primary causes of acute myocardial infarction (AMI-CS) and acute-on-chronic heart failure (HF-CS), has shown increased incidence and high mortality [1,2,3]. Despite advances in percutaneous coronary intervention (PCI) with early revascularization and the availability of mechanical circulatory support (MCS), the improvement in CS mortality has plateaued in the past 20 years since the SHOCK (SHould we emergently revascularize Occluded Coronaries for CS) trial [2,4,5]. There has been a decline in the incidence of AMI-CS and an increase in the prevalence of decompensated heart failure (HF) with shock due to non-ischemic causes as medical therapy evolves with changes in patient characteristics and comorbidities [5,6]. In a review study of patients with CS across 16 cardiac intensive care units in North America, 30% were due to AMI-CS. In contrast, 18% and 28% were related to ischemic and non-ischemic cardiomyopathy, respectively [5,7].

In addition to various causes and diverse clinical characteristics, CS involves a continuous progression of hemodynamic abnormalities from reversible myocardial dysfunction with straightforward hypotension to intractable shock with accumulated metabolic derangements and multiple organ failure [2,8,9]. In previous clinical trials, treating all patients with CS as a single group may have led to inconclusive results concerning prognosis and response to therapy, limiting the ability to develop evidence-based therapeutic approaches, especially in HF-CS. For example, although temporary MCS devices can effectively enhance cardiac output with hemodynamic improvement, thus, extending the therapeutic time window for recovery from myocardial and end-organ damage [2,10,11], MCS application has shown a limited mortality benefit in pooled analyses and randomized trials IABP-SHOCK I and IABP-SHOCK II [11,12,13]. However, IABP-SHOCK II trials enrolled CS patients who were all AMI, and only 15% of patients received an intra-aortic balloon pump (IABP) before reperfusion therapy [14], resulting in lower in-hospital mortality in several studies [15,16,17,18]. Nevertheless, MCS devices can still be helpful when applied correctly in selected patients according to risk profiles, optimal MCS initiation timing, and shock severity [2,19]. A meta-analysis of thirty-three studies encompassing 5204 CS patients showed that positioning Impella before starting PCI was associated with lower short-term mortality. In contrast, older age and severe comorbidities such as diabetes mellitus (DM) significantly reduced the benefit of MCS use in CS [19].

Therefore, it is imperative to develop treatment strategies based on risk stratification and validated prognostic scores in this group with high levels of heterogeneity, particularly for the implantation of MCS devices that are resource-intensive and invasive with a risk of complications.

The prognosis and hemodynamics of CS due to non-ischemic causes are poorly understood with few evidence-based treatments [2]. Therefore, it may not be appropriate to extrapolate the characteristics of patients with AMI-CS to those with CS due to non-ischemic causes in the previous prediction systems, such as CardShock and IABP-SHOCK II risk scores, which were derived primarily from myocardial infarction complicated by CS [20,21]. In an external validation study, the two scores showed modest prognostic accuracy in patients without acute coronary syndrome (AMI) (CardShock AUC 0.648, IABP-SHOCK II AUC 0.619, *p* = 0.31), a result that reflects the complexity of this population [20,21,22]. These studies developed scores in Western countries with limited Asian participants. The patients with AMI in Asian countries tend to be younger and have a higher burden of comorbidities, including diabetes, hypertension, and renal failure [23,24]. As AMI treatment advances, it may limit the validity of the previously established risk scores in contemporary patients.

This study aimed to describe the clinical characteristics of patients with all-cause CS in the emergency department (ED), identify risk factors, and establish a predictive model of short-term mortality risk.

## 2. Materials and Methods

### 2.1. Study Design and Inclusion Criteria

The institutional review board of Taichung Veterans General Hospital (TCVGH), Taichung, Taiwan, approved our study (CE22240B) following the ethical guidelines of the Declaration of Helsinki. However, the patients waived the informed consent due to the retrospective design.

We conducted this retrospective observational study in a tertiary care center in Taiwan (TCVGH) that receives ~65,000 ED visits and performs PCI for 1500 cases yearly. The clinical outcomes and risk factors for 30-day mortality were evaluated in patients over 18 years of age who presented with CS to the ED between 1 January 2014 and 31 December 2019. We excluded the patients who developed CS after admission due to the uncertain time intervals between CS detection and data collection. The criteria for CS included systolic blood pressure (SBP) < 90 mmHg for 30 min despite adequate fluid resuscitation or the need for inotropes or vasopressors to maintain SBP ≥ 90 mmHg and clinical signs of hypoperfusion (altered mental status, cold extremities, urine output < 0.5 mL/kg/h, serum lactate ≥ 2.0 mmol/L). The exclusion criteria included shock of non-cardiac origin, out-of-hospital cardiac arrest (OHCA), and arrhythmia as significant causes of hypotension. For diagnostic accuracy and primary data collection without disruption by the circulatory interruption, we excluded patients with in-hospital cardiac arrest (IHCA) in the ED without obtaining laboratory data or echocardiographic evaluation.

### 2.2. Data Collection and Definition

We extracted the data of demographics, underlying medical conditions, clinical manifestation, first values of biochemistry before intervention on the arrival of ED, echocardiography, angiography, treatment, and outcome from the electronic medical record. AMI-CS population included patients with ST-elevation myocardial (STEMI) and non-STEMI (NSTEMI).

We calculated the estimated glomerular filtration rate (eGFR) using the chronic kidney disease epidemiology collaboration (CKD-EPI) equation. According to the etiology of CS, we divided into the AMI-CS and HF-CS subgroups. We calculated the CardShock risk scores using each parameter. The use of inotropic and vasoactive agents and the indication for endotracheal intubation were according to the clinical conditions. A 24-h on-call intervention team performed primary PCI according to the door-to-balloon time protocol. The mode of primary PCI (the target lesion only or additional PCI for non-target lesions) was according to the vascular conditions. The diagnostic accuracy was independently verified by a chart review by a board-certified cardiologist and an emergency physician.

### 2.3. Study Outcomes

The primary outcome of all enrolled patients was all-cause mortality at 30 days.

### 2.4. Statistical Analysis

We expressed categorical variables as numbers and percentages and analyzed statistical differences using the Chi-square test (χ^2^ test). We expressed the continuous variables as mean and standard deviation (SD) or median and interquartile range. We used the student’s *t*-test or Mann–Whitney test to analyze statistical differences. The variables associated with mortality in the univariate Cox proportional hazards regression analyses (*p* < 0.05) were retained to enter a stepwise Cox multivariate analysis by which the remaining variables significantly associated with mortality constitute the score parameters. The continuous variables were further dichotomized with the optimal cut-off points defined using the Youden index. A restricted cubic spline plot was fitted with a Cox proportional hazard model adjusting for covariates to examine the nonlinear relationship between platelet (PLT) counts and the risk of 30-day mortality. The scoring system was determined based on the parameters’ respective hazard ratio (HR), assigning 1 or 2 points to each variable, and classified into three risk categories according to total scores. We assessed the discriminative ability of the risk prediction model in the area under the receiver operating characteristic curve (AUC) or the c-statistic. We calculated and plotted the population distribution and the mortality risk according to the cumulative points. We used the chi-square and Kaplan–Meier analyses with a pairwise log-rank test to compare the 30-day mortality rate. A two-sided *p* < 0.05 was regarded as statistically significant.

## 3. Results

### 3.1. Demographics and Clinical Characteristics

Table 1 compares the clinical characteristics of CS patients related to HF and AMI. Two hundred and twenty-five patients who presented to the ED with CS were enrolled. One hundred and fifty-nine patients (70.67%) were male, and the non-AMI causes were predominant (52.4%; *n* = 118). The mean age was 70.99 (62.52–80.8) years, and the AMI-CS subgroup was older. Regarding comorbidities, typical cardiovascular risk factors were common, and 82 patients (36.44%) had a history of coronary artery disease. Obstructive lung disease and atrial fibrillation were more prevalent in patients with HF-CS. The initial SBP was 82.86 mmHg, the diastolic blood pressure was 53.74 mmHg, and the heart rate was 89.44 beats per minute. One hundred and nine patients (48.44%) had acute pulmonary edema. More patients with AMI-CS presented conscious confusion than HF-CS (61.68% vs. 45.76%, *p* = 0.024). On average, the left ventricular ejection fraction (LVEF) was 30%, significantly higher in the AMI-CS subgroup (34% vs. 26.5%, *p* = 0.001). In general, 174 patients (77.33%) had LVEF < 40%, and 140 patients (62.22%) had moderate to severe mitral regurgitation. The mean serum lactate value was 2.99 mmol/L; more than 70% of patients had a lactate value > 2 mmol/L. Compared to HF-CS, patients with AMI-CS had higher white blood cell counts, PLT counts, CK-MB and glucose levels, and lower pH values. We found 53 patients (23.55%) with PLT counts < 155 × 10^3^/μL.

### 3.2. Management and Outcomes

A higher proportion of patients with AMI-CS received mechanical ventilation (70.09% vs. 51.69%, p = 0.007). The preferred inotrope and vasopressor for shock treatment were dopamine and norepinephrine. Nine patients with AMI-CS were treated conservatively with drugs alone, while ninety-eight received reperfusion therapy. Coronary angiography showed a three-vessel disease in 43 patients and a left-main culprit lesion in 20 patients. Fifty-five patients underwent IABP, which is more common in the AMI-CS subgroup (36.45% vs. 13.56%, p < 0.001). Fifty-seven patients experienced ventricular arrhythmias, and forty-eight had an IHCA. Of the 225 patients, 49 (21.78%) died in 30 days. Compared to HF-CS, patients with AMI-CS had higher CardShock scores (5.14 ± 1.50 vs. 3.97 ± 1.53, p < 0.001) and a more extended hospital stay (14 [9,10,11,12,13,14,15,16,17,18,19,20,21,22,23] vs. 11 [7–18.25] days, p = 0.004), but mortality at 30 days was numerically lower (15.89% vs. 27.12%, p = 0.061).

### 3.3. Multivariate Cox Regression Analysis to Identify Significant Variables

As shown in Table 2, after the multivariate analysis of the Cox model, four variables remained statistically significant, including PLT counts, LVEF < 40%, age, and lactate (PEAL). A decrement of 1000 PLT counts has an incremental mortality rate of 0.8% in overall cases (HR 1.008, 95% CI 1.004–1.012, p < 0.001). The mortality rate remained significant in the analysis of subgroups, AMI-CS with HR 1.009, 95% CI 1.002–1.017, p = 0.01, and HF-CS with HR 1.006, 95% CI 1.001–1.011, p = 0.014.

### 3.4. Nonlinear Relationship between Platelet Counts and 30-Day Mortality

**Figure 1:**
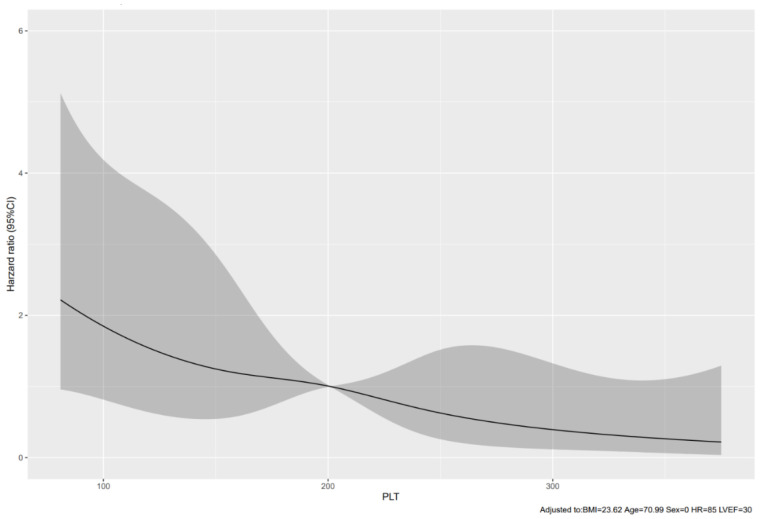
The restricted cubic spline plot shows hazard ratios with 95% confidence intervals for mortality in 30 days according to platelet counts. The plot was fitted with the Cox proportional hazards model, adjusting for age, sex, body mass index, heart rate, and left ventricular ejection fraction. The cut-off point of platelet counts was 200 × 10^3^/μL. The shades of grey color presented the 95% confidence intervals of hazard ratios.

To assess the nonlinear relationship between PLT counts and mortality, a restrictive cubic spline plot was fitted with a Cox proportional risk model with adjustments for body mass index, age, sex, heart rate, and LVEF. As shown in Figure 1, the decrease in PLT counts was significantly associated with an increased 30-day mortality risk, with an inflection point at 200 × 10^3^/μL of PLT counts. We determined the second cut-off point of PLT counts < 155 × 10^3^/μL by the Youden index.

### 3.5. Determine the Cutoff Points of the Predictors and the Risk Scoring System

Age > 71 years and lactate > 2.7 mmol/L were determined as the cutoff points using the Youden index. Each predictor was assigned a score of 0 or 1 depending on the HR of the dichotomized variables, as shown in Table 3. The reflection point of 200 × 10^3^/μL in the restrictive cubic spline plot was used as the first cutoff point for PLT counts. A score of 2 was assigned for PLT counts less than 155 × 10^3^/μL. We developed the PEAL score risk model (platelet, LVEF, age, and lactate) to predict the 30-day mortality risk in patients with CS in the ED, as shown in Table 4.

### 3.6. Receiver Operating Characteristic Curve (ROC) of the Risk Score

**Figure 2:**
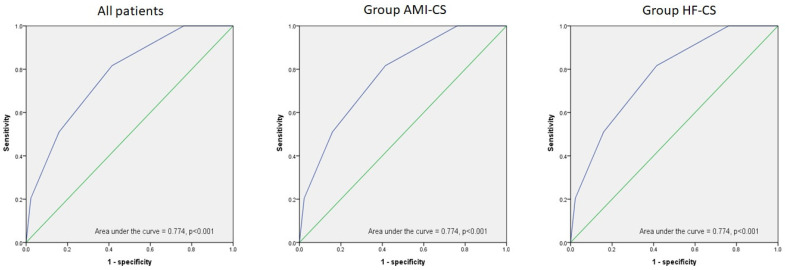
The receiver operating characteristic curve (ROC) of the risk score to predict mortality risk at 30 days in all patients with CS (*n* = 225) (**A**). The ROC of the risk score to predict mortality risk at 30 days in the AMI-CS subgroup (*n* = 107) (**B**). The ROC of the risk score to predict mortality risk at 30 days in the HF-CS subgroup (*n* = 118) (**C**). The blue lines indicated the curves in the AUC of the ROC for all patients (**A**), ACS patients (**B**), and Non-ACS patients (**C**). The green lines presented the baseline in the AUC of the ROC.

Table 5 shows that this risk-scoring model has good predictive power for 30-day mortality risk in the AMI-CS and HF-CS subgroups (AUC of 0.774 for all, AUC of 0.781 for AMI-CS, and AUC of 0.759 for HF-CS), regarding the ROC curves in Figure 2.

### 3.7. Distribution of the Risk Score and Observed Mortality

**Figure 3:**
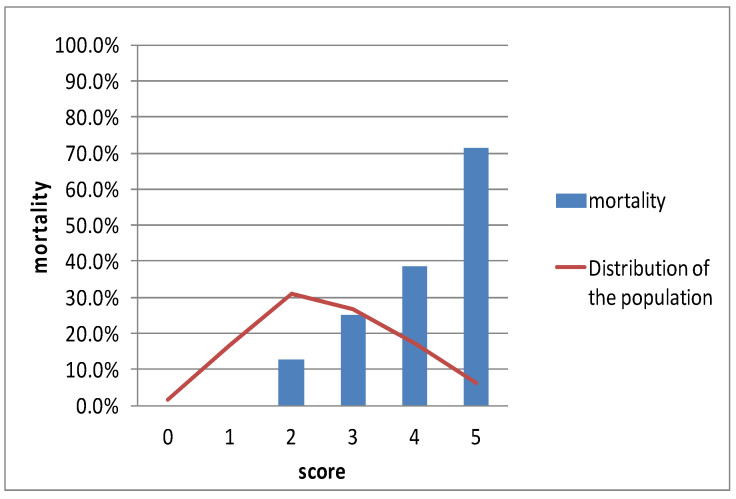
Distribution of the study population (red line) and mortality rate observed at 30 days (%; blue bars) across the cumulative points of the risk score.

Figure 3 shows the distribution of the study population and the stepwise increase in observed mortality within 30 days as the risk scores increase. There were no deaths in the 0 and 1 scores. The 30-day mortality rates for scores 2, 3, 4, and 5 were 13%, 25%, 38%, and 71%, respectively. The blue lines indicated the curves in the AUC of the ROC for all patients (A), ACS patients (B), and Non-ACS patients (C). The green lines presented the baseline in the AUC of the ROC.

### 3.8. Cumulative Mortality according to Score Categories Using the Kaplan–Meier Method

**Figure 4:**
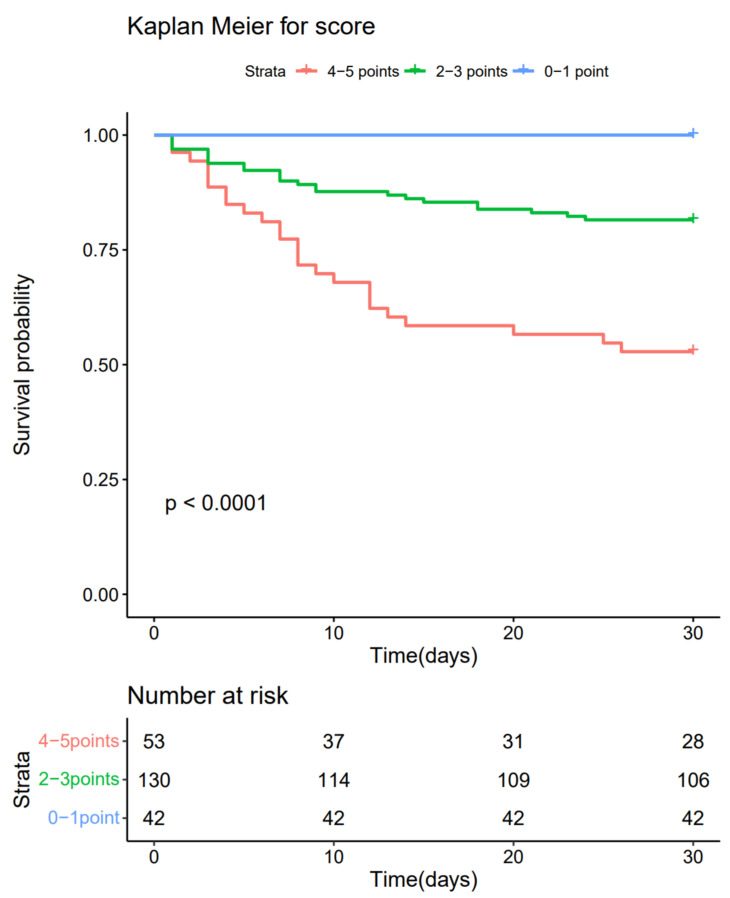
Kaplan–Meier survival curve for 30-day mortality according to score categories with pair-wise comparisons by log-rank test.

According to the risk scores, 42 patients (18.7%) were at low risk (0–1), 130 patients (57.8%) were at moderate risk (2–3), and 53 patients (23.5%) were in the high-risk group (4–5). The Kaplan–Meier survival curves showed significant differences in cumulative mortality for 30 days between the three subgroups, compared by log-rank test (*p* < 0.001 for 0–1 vs. 2–3; *p* < 0.001 for 2–3 vs. 4–5; and *p* < 0.001 for 0–1 vs. 4–5) (Figure 4).

## 4. Discussion

The study described the clinical characteristics and real-world practices of 225 patients with all-cause CS in the ED of a tertiary care hospital in Taiwan. With a higher proportion of patients with CS not related to AMI (HF-CS 52.4% vs. AMI-CS 47.6%), the study revealed more information on the HF-CS subgroup, which had been less addressed in the literature. Therefore, we established a predictive model of 30-day mortality for CS based on four significant variables in multivariate analyses using the Cox model, including PLT counts < 200 × 10^3^/μL, LVEF < 40%, age > 71 years, and lactate > 2.7 mmol/L. The model showed good predictive power with an AUC of 0.774 with 95% CI (0.705–0.843) for all patients, 0.781 (0.678–0.883) for AMI-CS, and 0.759 (0.662–0.855) for HF-CS, all *p* < 0.001. Furthermore, the study showed that a decrement of 1000/μL in PLT counts was associated with an increment of 0.8% in cumulative 30-day mortality risk of CS, even within the normal range of PLT counts (HR 1.008, 95% CI 1.004–1.012, *p* < 0.001). This association remained significant when analyzed in the AMI-CS and HF-CS subgroups. AMI-CS had an HR of 1.009 with 95% CI 1.002–1.017, *p* = 0.01, and HF-CS had an HR of 1.006, 95% CI 1.001–1.011, *p* = 0.014. This model is the first risk score incorporating the number of PLT counts at presentation to predict short-term mortality in patients with AMI-CS and HF-CS.

The study focused on patients with CS in the ED (primary CS). At the same time, most of the criteria in the previous trials were carried out to predict the development of CS after hospital admission in patients with AMI (secondary CS) [25,26,27,28,29]. The recent studies have shown different trends in the incidence and mortality between primary and secondary CS. In Western countries, a stable mortality rate was observed for secondary CS (from 64.5% to 69.1%, *p* = 0.731), while the mortality rate for primary CS decreased (from 60% to 38%, *p* = 0.038). There was also a trend toward a decrease in secondary CS during hospitalization [30]. It was attributed to a timely PCI, which limits the size of the infarction and reduces subsequent complications. With the increasing population of advanced HF after AMI, CS on admission (primary CS) now accounts for most CS cases. The exclusive enrollment of primary CS cases and the exclusion of patients with OHCA and non-survivors of IHCA in the ED may have led to a lower 30-day mortality rate (21%) in this study. More importantly, mandatory health insurance and widespread hospital networks for AMI in Taiwan could reduce the time interval between the onset and coronary revascularization and decrease the mortality rate in AMI-CS by early interruption of the devastating shock spiral from isolated myocardial dysfunction to progressive multiorgan failure [8,9].

Compared to the CardShock study concerning shock severity, the study population showed comparable clinical characteristics, including age, the prevalence of comorbidities, LVEF, lactate, and eGFR, as well as a similar reperfusion rate (91% vs. 90%) in AMI-CS [20]. Three predictors in the CardShock score did not show a significant association with mortality in the study, including the etiology of AMI, the eGFR, and conscious confusion at presentation. There are some reasons for this. The study population showed a higher prevalence of chronic kidney disease (CKD) and worse mean renal function compared to the CardShock study. The mean eGFR was 42 (range of 25–65) in our study and 61 (range of 41–87) in the CardShock study. The prevalence of CKD was 20.44% in our study and 11% in the CardShock study. Consequently, using eGFR as a biomarker of end-organ dysfunction could be confounding by different baseline renal functions, particularly in areas with prevalent CKD. Furthermore, the assessment of confusion depends on the physician’s subjective judgment, and the level of consciousness is susceptible to using oxygen and vasopressors or sedative medications during mechanical ventilation.

Comparing with HF-CS, AMI-CS is generally more severe with a higher SCAI shock stage despite similar hemodynamics and better LVEF [31]. In our study, AMI-CS showed higher LVEF, older, confusion, mechanical ventilation, and IABP support than those with HF-CS. Additionally, although statistically insignificant, more patients with AMI-CS experienced ventricular arrhythmias and cardiovascular collapse. Patients with AMI also showed drastic features in laboratory data, including higher white blood cell and glucose levels and more severe acidemia in arterial blood gases. Despite a more serious clinical presentation, patients with AMI-CS had a lower mortality rate than those with HF-CS, which differed from most previous studies. Timely transport and revascularization in Taiwan reduced the damaged area of the myocardium and were supposed to be the main reason for the lower mortality. Therefore, the etiology of AMI should be considered a modifiable factor rather than a constant predictor of poor outcomes in CS.

In our study, four variables showed a significant relationship with mortality in CS. However, too many predictors can be prone to overfitting and limited generalization to external populations in CS with high heterogeneity. Aging has been the most common patient-related risk factor in published prognostic scores for CS [32], possibly due to limited physiological reserve and compensatory capacity. When applying MCS in CS, older age was also significantly associated with more adverse clinical course and higher complication rates, resulting in a narrow therapeutic range for clinical physicians [19].

Lactate was a standard marker to predict the clinical outcome of CS [33,34]. Hyperlactatemia reflects impaired tissue perfusion, and cumulative cellular metabolic derangements have proven to be associated with increased mortality in the literature. In the current study, an increase of each mmol/L in lactate was associated with an adjusted HR of 1.1 for mortality at 30 days. Both older age and lactate were predictive variables in the CardShock and IABP-SHOCK II risk scores [20,21]. As a continuous variable, LVEF did not show a significant association with mortality in this study, possibly because the shock severity is related not only to reduce cardiac output and primitive LV function before the myocardial injury but also to maladaptive circulatory compensation. In the study, an LVEF < 40% was associated with increased mortality risk in CS, compatible with the results of several studies [20,35,36].

Thrombocytopenia is associated with an increased mortality rate in patients who are admitted to intensive care units [37] and in multiple cardiovascular diseases, including AMI, HF, and transcatheter aortic valve implantation (TAVI) [38,39,40,41,42]. In patients with AMI, studies have indicated an association between thrombocytopenia and adverse cardiovascular outcomes, including all-cause or cardiovascular death, myocardial infarction, and target lesion revascularization [39,41,43,44,45,46]. A multicenter retrospective study in 1907 patients with HFrEF (LVEF < 40%) showed higher all-cause mortality in patients combined with moderate/severe thrombocytopenia (PLT counts < 100 × 10^3^/μL) compared to normal/mild thrombocytopenia (HR 1.84, 95% CI 1.33–2.56, *p* < 0.001) [39]. In a study on patients with AMI randomly assigned to unfractionated heparin or hirudin, Eikelboom et al. demonstrated a similar relationship between thrombocytopenia and adverse non-hemorrhagic outcomes in patients with AMI. They proposed that excessive PLT activation may lead to PLT consumption and the further deterioration of coronary ischemia [44]. Moreover, several studies have reported that a reduced number of PLT counts is associated with the increased mortality rate in CS with VA-ECMO and IABP institutions, with the primary mechanism involving mechanical consumption of circulating PLT counts [47,48,49,50,51].

However, CardShock and IABP-SHOCK II risk scores did not include PLT counts as a predictor [20,21], and there is a lack of research linking PLT counts with the prognosis of CS. A recent multicenter study in South Korea, investigating a total of 1202 patients with all-cause CS from 2014 to 2018, concluded that a decrease in PLT counts at the presentation of CS was associated with increased all-cause mortality at 30 days in the multivariate Cox model (a decrease of 1000/μL in PLT counts, HR 1.002, 95% CI 1.000–1.030, *p* = 0.021) [52], consistent with the finding in the present study, with an identical inflection point at 200 × 10^3^/μL in restrictive cubic spline plots. Furthermore, the study showed an incidence of thrombocytopenia of 20%, close to the 23% in the present study. Consequently, PLT counts could be underestimated and not adequately evaluated regarding their predictive value for the prognosis of CS.

The pathophysiological mechanisms underlying the occurrence of thrombocytopenia in CS have yet to be fully elucidated. The mean PLT volume and the PLT surface P-selectin were used as PLT activation markers in different studies and were found to be at higher levels in patients with acute decompensated HF than in stable HF [53,54,55,56,57,58]. Therefore, low PLT counts may be due to abnormal PLT activation and the subsequent destruction during the worsening status of HF. Hemodynamic instability can lead to stagnant blood flow, predisposing to PLT aggregation. In an advanced shock state, maladaptive compensatory mechanisms involving systemic inflammation, increased catecholamines, and renin–angiotensin system activation can contribute to PLT overactivation and consumption [59,60,61,62]. AMI-CS can deteriorate drastically with sharply elevated lactate levels. As the shock progresses, a decrease in PLT counts may reflect maladaptive systemic inflammation and neurohormonal compensatory responses, typical of decompensated chronic HF, in which PLT counts participate by acting as inflammatory mediators [9,63].

Based on four routinely available metrics, the current score has the advantages of being easy to calculate, allowing early prognosis prediction, and facilitating individualized therapeutic strategies from non-invasive medical treatment (Score 0–1) to maximum therapeutic options, including MCS implants for those who may have the most incredible benefits (Score 2–4) or providing palliative care for futile patients (Score 5). Due to the aging population with an increased burden of comorbidities, identifying futile groups is vital in areas with limited medical resources in the post-pandemic era.

## 5. Limitation

There are several limitations to be acknowledged in the study. First, this is a retrospective study of a single medical center with potentially unmeasured confounders and incomplete data; therefore, the causality between PLT counts and the outcome could not be established, and the underlying mechanism warrants further investigation. Although the diagnostic accuracy was verified by reviewing the charts, only clinical, biochemical, and echocardiographic data were universally available without providing hemodynamic measurements to clarify pure cardiogenic or mixed shock states. Concerning MCS devices, IABP was used exclusively in the study because the National Health Insurance did not cover the payment of Impella in Taiwan, which might limit the extension of study results. In addition to the limited sample size of a single database, the inclusion of patients with CS in the ED may have incurred selection bias. However, given the high heterogeneity of CS populations, it is unlikely that any single-derivation cohort will fully reflect an external population. Since the data were collected before starting any treatment, heparin-induced or mechanically disrupted thrombocytopenia with MCS could be excluded. Moreover, an extended 95% CI in the restricted cubic spline plot may indicate a more significant margin of error and a less precise estimate, partly due to the small sample size. Finally, the longitudinal follow-up on the PLT counts was lacking, which can provide more evidence of prognostic strength by relating to adverse events and hospital course.

## 6. Conclusions

Based on four parameters, platelet counts, LVEF, age, and lactate (PEAL), this pre-diction model showed a good predictive performance for all-cause mortality at 30 days in all patients and the AMI-CS and HF-CS subgroups. The restrictive cubic spline plot showed a significantly negative correlation between initial PLT counts and 30-day mortality in AMI-CS and HF-CS subgroups. The feasibility of trending serial PLT counts as clinical markers related to the severity and outcome of CS deserves further evaluation in more extensive studies.

## Figures and Tables

**Table 1:** Characteristics, clinical manifestations, laboratory data, echocardiography, shock management, reperfusion therapy, outcomes, and complications of patients with HF-CS (*n* = 118, 52.4%) and AMI-CS (*n* = 107, 47.6%).

Baseline Characteristics	All, *n* = 225 (100%)	HF-CS, *n* = 118 (52.4%)	AMI-CS, *n* = 107 (47.6%)	*p*-Value
Age (years)	70.99 (62.52–80.8)	68.68 (55.17–78.36)	74.16 (65.26–83.52)	0.004 **
Male	159 (70.67%)	79 (66.95%)	80 (74.77%)	0.254
Body mass index	24.02 ± 4.66	23.43 ± 4.10	24.66 ± 5.13	0.072
Coronary artery disease	82 (36.44%)	45 (38.14%)	37 (34.58%)	0.678
Prior PCI	47 (20.89%)	19 (16.10%)	28 (26.17%)	0.091
Prior CABG	24 (10.67%)	13 (11.02%)	11 (10.28%)	1.000
Diabetes mellitus	86 (38.22%)	44 (37.29%)	42 (39.25%)	0.869
Hypertension	108 (48.00%)	54 (45.76%)	54 (50.47%)	0.567
Dyslipidemia	69 (30.67%)	32 (27.12%)	37 (34.58%)	0.286
Asthma/COPD	42 (18.67%)	31 (26.27%)	11 (10.28%)	0.004 *
Old CVA	18 (8.00%)	8 (6.78%)	10 (9.35%)	0.644
Peripheral artery disease	12 (5.33%)	5 (4.24%)	7 (6.54%)	0.637
Atrial fibrillation	63 (28.00%)	49 (41.53%)	14 (13.08%)	<0.001 **
Renal insufficiency	46 (20.44%)	28 (23.73%)	18 (16.82%)	0.264
End-stage renal disease	3 (1.33%)	2 (1.69%)	1 (0.93%)	1.000
Hypothyroidism	17 (7.56%)	12 (10.17%)	5 (4.67%)	0.192
Clinical presentation				
SBP (mm Hg)	82.86 ± 16.67	82.01 ± 14.72	83.80 ± 18.60	0.962
DBP (mm Hg)	53.74 ± 12.75	54.81 ± 11.61	52.56 ± 13.85	0.077
Heart rate (b.p.m.)	89.44 ± 25.25	92.79 ± 25.79	85.76 ± 24.22	0.060
Confusion	120 (53.33%)	54 (45.76%)	66 (61.68%)	0.024 *
Acute pulmonary edema	109 (48.44%)	58 (49.15%)	51 (47.66%)	0.929
Laboratory data				
WBC counts (/μL)	11,106.71 ± 5239.41	9703.39 ± 4414.11	12,654.30 ± 5647.51	<0.001 **
Platelet counts (10^3^/μL)	210.01 ± 85.85	185.58 ± 73.37	236.95 ± 90.76	<0.001 **
Hemoglobin (g/dL)	12.35 ± 2.51	12.54 ± 2.15	12.14 ± 2.85	0.232
Total bilirubin (mg/dL)	0.80 (0.5–1.6)	1.20 (0.7–2.5)	0.60 (0.4–0.9)	<0.001 **
Glucose (mg/dL)	158 (118–240)	140 (111–185)	200 (144–260)	<0.001 **
eGFR (mL/min/1.73 m2)	42.13 (24.65–64.53)	45.26 (26.45–66.29)	40.60 (20.08–63.48)	0.152
CKMB (U/L)	11.00 (7–20)	9.00 (6–15.25)	14.00 (8–31)	<0.001 **
NT-proBNP (pg/mL)	10,010 (3993–26,700)	9993 (4383–24,037)	10,400 (3809–33,250)	0.878
pH	7.34 (7.26–7.39)	7.36 (7.29–7.4)	7.33 (7.24–7.38)	0.005 **
HCO_3_^−^ (mmol/L)	21.90 (18.8–26)	22.90 (19.5–27.3)	21.00 (18.25–24.9)	0.012 *
Lactate (mmol/L)	2.99 (1.8–5.01)	2.98 (1.79–5.89)	2.99 (1.79–4.27)	0.697
Lactate > 2 mmol/L	160 (71.11%)	83 (70.34%)	77 (71.96%)	0.904
Echocardiography				
LVEF (%)	30.00(20–39)	26.50(18–37)	34.00(25–41)	0.001 **
LVEF < 40%	174(77.33%)	101(85.59%)	73(68.22%)	0.003 **
Pulmonary hypertension	98(43.56%)	60(50.85%)	38(35.51%)	0.029 *
AR (moderate or severe)	66(29.33%)	37(31.36%)	29(27.10%)	0.580
MR (moderate or severe)	140(62.22%)	77(65.25%)	63(58.88%)	0.397
Vasopressors				0.003 **
Dobutamine	21 (9.33%)	14 (11.86%)	7 (6.54%)	
Dopamine	160 (71.11%)	91 (77.12%)	69 (64.49%)	
Norepinephrine	40 (17.78%)	13 (11.02%)	27 (25.23%)	
Epinephrine	4 (1.78%)	0 (0%)	4 (3.74%)	
Shock management				
Invasive MV	136 (60.44%)	61 (51.69%)	75 (70.09%)	0.007 **
IABP	55 (24.44%)	16 (13.56%)	39 (36.45%)	<0.001 **
ECMO	14 (6.22%)	6 (5.08%)	8 (7.48%)	0.642
Outcomes/Complications				
CardShock risk score	4.52 ± 1.62	3.97 ± 1.53	5.14 ± 1.50	<0.001 **
In-hospital cardiac arrest	48 (21.33%)	20 (16.95%)	28 (26.17%)	0.128
Ventricular arrhythmias	57 (25.33%)	25 (21.19%)	32 (29.91%)	0.178
30-day mortality	49 (21.78%)	32 (27.12%)	17 (15.89%)	0.061
Length of stay (days)	13 (8–22)	11 (7–18.25)	14 (9–23)	0.004 **

Chi–squared test. Mann–Whitney U-test.* *p* < 0.05, ** *p* < 0.01, statistically significant. The continuous data are expressed as mean ± SD or median (IQR); the categorical data are expressed as number and percentage. CS related to acute myocardial infarction, AMI-CS; aortic regurgitation, AR; beats per minute, b.p.m.; coronary artery bypass graft, CABG; diastolic blood pressure, DBP; extracorporeal membrane oxygenation, ECMO; acute-on-chronic heart failure with CS, HF-CS; intra-aortic balloon pump, IABP; left ventricular ejection fraction, LVEF; mitral regurgitation, MR; percutaneous coronary intervention, PCI; systolic blood pressure, SBP; mechanical ventilation, MV.

**Table 2:** Results of the multivariate Cox regression analysis.

Variables	Cox Multivariable Model		
	Hazard Ratio	95% CI	*p*-Value
Platelet (counts/μL)	1.008	1.004–1.012	<0.001 **
LVEF (%)	2.67	1.05–6.80	0.040 *
Age (years)	1.02	1.00–1.05	0.018 *
Lactate (mmol/L)	1.10	1.03–1.18	0.007 **

Cox regression analysis. * *p* < 0.05, ** *p* < 0.01, statistically significant. Left ventricular ejection fraction, LVEF.

**Table 3:** Hazard ratios of dichotomized variables associated with 30-day mortality.

Variables	Hazard Ratio	95% CI	*p*-Value
Platelet counts < 200 (×10^3^/μL)	2.574	1.379–4.805	0.003 **
Age > 71 (years)	2.452	1.327–4.531	0.004 **
LVEF < 40 (%)	2.613	1.020–6.692	0.045 *
Lactate > 2.7 (mmol/L)	1.967	1.069–3.620	0.030 *

Cox regression analysis. * *p* < 0.05, ** *p* < 0.01, statistically significant. Left ventricular ejection fraction, LVEF.

**Table 4:** Risk scoring model for the prediction of 30-day mortality in CS.

Variables		PEAL Score (Maximum 5 Points)
Platelet counts (×10^3^/μL)	>200	0
	155–200	1
	<155	2
LVEF < 40 (%)		1
Age > 71 (years)		1
Lactate > 2.7 (mmol/L)		1

Optimal cut-off points determined by Youden index. Left ventricular ejection fraction, LVEF; Platelet, LVEF, Age, Lactate, PEAL.

**Table 5:** The area under the curve (AUC) of the receiver operating characteristic curve (ROC) for the PEAL score.

PEAL Score	AUC of ROC	95% CI	*p*-Value
All, *n* = 225	0.774	0.705–0.843	<0.001 **
AMI-CS, *n* = 107	0.781	0.678–0.883	<0.001 **
HF-CS, *n* = 118	0.759	0.662–0.855	<0.001 **

Chi–squared test. ** *p* < 0.01, statistically significant. CS related to acute myocardial infarction, AMI-CS; acute-on-chronic heart failure with CS, HF-CS.

## Data Availability

Readers can access the data and material supporting the study’s conclusions by contacting Sung-Yuan Hu at song9168@pie.com.tw.
